# The Role of Innervation in Ocular Surface Homeostasis, Tissue Remodeling Following Nerve Injury, and the Therapeutic Potential of Hemocomponents in Neuronal and Cicatricial Pathologies †

**DOI:** 10.3390/jcm15052026

**Published:** 2026-03-06

**Authors:** Giulio Ferrari, Silvia Odorici, Matteo Menean, Antonio Di Zazzo, Piera Versura, Alessandra Micera

**Affiliations:** 1 Vita-Salute San Raffaele University, Department of Ophthalmology, 20132 Milan, Italy; ferrari.giulio@hsr.it; 2 IRCCS San Raffaele Scientific Institute, Division of Neuroscience, Cornea and Ocular Surface Disease Unit, Eye Repair Lab, 20132 Milan, Italy; menean.matteo@hsr.it; 3 Ophthalmology Unit, DIMEC, Alma Mater Studiorum Università di Bologna, 40138 Bologna, Italy; silvia.odorici2@studio.unibo.it; 4 Oftalmologia, Università Campus Biomedico—Centro Malattie Rare Corneali—Fondazione Policlinico Campus Biomedico, 00128 Rome, Italy; a.dizazzo@unicampus.it; 5 IRCCS Azienda Ospedaliero-Universitaria di Bologna, 40138 Bologna, Italy; 6 Research and Development Laboratory for Biochemical, Molecular and Cellular Applications in Ophthalmological Science, IRCCS-Fondazione Bietti, 00184 Rome, Italy; alessandra.micera@fondazionebietti.it

**Keywords:** ocular surface, corneal innervation, neurogenic inflammation, tissue remodeling, neurotrophic keratopathy, blood-derived eye drops, eye drops of human origin

## Abstract

The ocular surface is a neuro–epithelial–immune unit in which corneal innervation is essential for maintaining tissue integrity and visual function. Sensory nerves regulate reflex tearing and blinking, provide trophic support, and modulate local immune responses. Nerve injury resulting from trauma, surgery, infection, systemic disease, or chronic inflammation disrupts epithelial homeostasis and may lead to neurotrophic keratopathy, neuropathic pain, and pathological remodeling. Beyond classical neurotrophic disease, nerve dysfunction contributes to severe dry eye and immune-mediated cicatricial disorders. Depending on the neuro-inflammatory context, remodeling may evolve toward stromal thinning, as in keratoconus, or progressive fibrosis, as in ocular cicatricial pemphigoid. Blood-derived eye drops, including serum- and platelet-based formulations, represent biologically active therapies that support epithelial repair and nerve regeneration, although greater standardization is needed.

## 1. Conceptual Premises

The ocular surface represents a highly specialized and dynamic biological system in which epithelial cells, sensory nerves, immune components, and tear film constituents interact to preserve tissue integrity and visual function [1,2]. The dense sensory innervation of the cornea plays a central role not only in nociception but also in maintaining epithelial homeostasis, regulating tear secretion, and coordinating wound healing through the release of neuropeptides and growth factors [3].

Disruption of this finely regulated neuroepithelial unit—whether secondary to trauma, surgery, inflammation, infection, or chronic ocular surface disease—can profoundly impair tissue homeostasis. Loss or dysfunction of corneal nerves results in altered epithelial turnover, reduced trophic support, and impaired regenerative capacity, ultimately leading to persistent epithelial defects, neurotrophic keratopathy, and progressive cicatricial remodeling [1]. Increasing evidence suggests that these mechanisms extend beyond classical neurotrophic disease and contribute to the pathophysiology of severe dry eye disease, immune-mediated ocular surface disorders, and post-surgical complications [4,5,6].

A disrupted balance between tissue homeostasis and para-inflammation is increasingly recognized as a key driver of ocular surface remodeling. Although initially protective, this response may become dysregulated, leading to fibrosis, loss of stromal architecture, and functional impairment. Recent studies have highlighted the role of corneal innervation and neurogenic inflammation in these processes, particularly in keratoconus and ocular cicatricial pemphigoid—two paradigmatic disorders characterized by opposite patterns of tissue remodeling—as well as stromal thinning in keratoconus and fibrotic matrix accumulation in ocular cicatricial pemphigoid.

In recent years, growing interest has focused on biological therapies capable of restoring the complex microenvironment required for epithelial and neural recovery. Among these, blood-derived eye drops—including autologous serum, platelet-rich plasma, platelet lysate, and cord blood–derived preparations—have emerged as promising therapeutic tools. These products contain a broad spectrum of growth factors, cytokines, neurotrophins, and extracellular vesicles that closely resemble the biochemical composition of natural tears and are known to support epithelial proliferation, modulate inflammation, and promote nerve regeneration [7].

Experimental and clinical studies have demonstrated that blood-derived products can enhance corneal epithelial healing, improve nerve morphology and function, and alleviate symptoms in patients with neurotrophic and cicatricial ocular surface disease. However, their clinical application remains heterogeneous, with variability in preparation protocols, dosing regimens, and indications, highlighting the need for better standardization and mechanistic understanding.

This narrative review aims to provide an updated overview of the role of corneal innervation in ocular surface homeostasis, to examine the pathophysiological consequences of nerve injury, and to critically discuss the therapeutic potential of blood-derived eye drops in neuronal and cicatricial ocular surface disorders. By integrating experimental evidence with clinical experience, this review seeks to clarify current knowledge gaps and support a more rational and targeted use of these biologically active therapies in clinical practice.

## 2. The Role of Innervation in Ocular Surface Homeostasis

### 2.1. Introduction

The ocular surface is the most densely innervated tissue in the human body. This intricated network includes sensory, motor, and autonomic nerve fibers. It guarantees complex functions, which are crucial for corneal homeostasis and consequently vision preservation [8,9]. The complex interplay at the base of these networks is of utmost importance for innate reflexes (like blinking and tearing) [10]. Additionally, the neural network orchestrates the metabolic support and immune regulation [11]. Recent advances in imaging, namely in vivo confocal microscopy (IVCM), and advanced bio-molecular analyses, have revolutionized our understanding of ocular innervation (not only in healthy eyes, but also in pathological settings), setting the base for in-depth comprehension of several conditions, such as neurotrophic keratopathy (NK) and chronic neuropathic pain [12,13,14].

Specifically, disruption of these nerve fibers, which can be promoted by disease, trauma, metabolic conditions, or aging, can precipitate a cascade of degenerative changes, culminating in vision loss [15]. The aim of the review is to review our contemporary knowledge from key studies and clinical practice, detailing the anatomy of ocular nerves and their physiological roles, including pathological consequences and consequent tissue remodeling, and then focusing on hemocomponents as new therapeutic innovations.

### 2.2. Anatomy of Ocular Innervation

#### 2.2.1. Corneal Innervation

The cornea’s nerve supply primarily arises from the ophthalmic division (V1) of the trigeminal nerve. The ophthalmic branch passes through the superior orbital fissure and its branches. It innervates the cornea with the long ciliary nerve, a branch of the nasociliary nerve. An average of 60–80 stromal nerve trunks enter the cornea through the peripheral limbus. These nerves create a midstromal network, which subsequently branches into the subepithelial region, directly beneath Bowman’s layer, thus creating the subbasal nerve plexus. This plexus radiates toward the corneal surface in a spiral vortex-like pattern. These radiations are responsible for rapidly detecting mechanical, thermal, and chemical stimuli essential to corneal homeostasis and protection [16,17].

Neuro-anatomical studies report two main types of corneal sensory fibers: myelinated A-delta fibers and unmyelinated C fibers. The first mediate acute tactile and sharp pain sensations, while the latter respond to temperature extremes and chemical irritants. This densely represented network contains an abundance of neuromediator-releasing terminals, as it has been proven by immunostaining for neural markers, such as protein gene product 9.5 (PGP9.5) and calcitonin gene-related peptide (CGRP) [16,17]. There is also a topographical heterogeneity inside the cornea itself: the central cornea exhibits the highest density, while the periphery, though less concentrated, maintains a similar function to prevent focal hypersensitivity [18,19]. Additionally, a similar molecular gradient of growth factors (nerve growth factor—NGF, and brain-derived neurotrophic factor—BDNF) guides corneal embryogenesis, postnatal maturation, but also epithelial healing and wound closure [20].

#### 2.2.2. Conjunctival and Scleral Innervation

The sensory innervation of the conjunctiva comes from nasociliary and lacrimal branches of the ophthalmic division of the trigeminal nerve. Of note, the conjunctiva has a much lower density of innervation compared to the cornea. Imaging of conjunctival nerves using IVCM suggests regional specialization and heterogeneity between the bulbar and tarsal conjunctiva. Namely, the bulbar conjunctiva has more nerve endings compared to the tarsal one [8]. The conjunctival sensory nerve’s function is not limited to the detection of environmental irritants and foreign bodies, but also modulates tear and mucous secretion through interaction with the meibomian gland and regulates local immune activation. During allergic or inflammatory episodes, conjunctival nerve fibers interact with mast cells and dendritic cells, amplifying the immune response by releasing substance P and other peptides [21,22,23]. Ultimately, the sclera is innervated by the long posterior ciliary nerves. They contribute to deep and dull pain, and they additionally support vascular autoregulation during inflammation [24].

#### 2.2.3. Eyelid and Periocular Region Innervation

Eyelid innervation encompasses sensory input from the ophthalmic (through the supraorbital and supratrochlear nerves) and maxillary (through the infraorbital nerve) branches of the trigeminal nerve, supporting fine tactile sensitivity, pain detection, and participating in protective reflexes. Motor function primarily consists of eyelid closure and blinking, and it is governed by the facial nerve (VII) through the orbicularis oculi and levator palpebrae muscles [25,26]. Damage to the facial nerve may cause ptosis and compromised blink reflex, with consequent high-risk exposure keratopathy and related infections [26]. Autonomic regulation primarily controls the tone of the Muller’s muscle, but also smooth muscle components of the lid and the vasculature. It is mediated by sympathetic fibers arising from the superior cervical ganglion [27,28,29]. Parasympathetic innervation is much less robust and contributes to meibomian gland secretion regulation [30].

### 2.3. Physiological Functions of Ocular Nerves

The physiological impact of ocular innervation is profound, and it is not limited to basic sensation and muscle activation. Sensory, motor, and autonomic nerves interact together, and they guarantee ocular surface homeostasis, with corneal nerves playing a pivotal role [8].

#### 2.3.1. Corneal Reflex

The corneal reflex represents one of the fastest defensive mechanisms. When the corneal surface is stimulated (either by touch, particulate matter, or chemical agents), afferent stimuli reach the spinal trigeminal nucleus, initiating an immediate bilateral blink via facial nerve efferents [31]. Blink onset takes only 50 milliseconds, as confirmed by latency studies [32]. This ancestral reflex protects the eye, washes away foreign bodies, and minimizes injury risk.

The tear reflex has a similar function: a stimulation of the cornea or conjunctiva triggers the lacrimal gland through parasympathetic secretomotor pathways and promotes tear secretion. This mechanism allows hydration maintenance and restores epithelial integrity. Blinking, combined with tear production, guarantees a safe mechanism for ocular pH regulation and limits microbial pathogen colonization [33].

#### 2.3.2. Trophic Support and Tear Regulation

Nerve terminals in the corneal and conjunctival epithelium allow the secretion of important neuromediators (encompassing substance P and CGRP, as well as neurotrophins like NGF and BDNF). These molecules play pivotal roles by promoting epithelial cell proliferation and migration [16,17,20]. Additionally, they upregulate protective gene programs and allow immune cell migration to sites of injury for healing. CGRP has anti-inflammatory effects, while substance P favors wound healing and immune cell recruiting. Absence or reduction in these molecules is usually seen in denervation, and it leads to compromised epithelial health, culminating in persistent epithelial defects with associated delayed repair [23,34].

The lacrimal and meibomian glands also respond to neural stimulation by adjusting mucous and lipid balance, essential for tear film stability. Neural signaling also modulates secretory rates and influences tissue barrier function [33].

#### 2.3.3. Nociception and Pain Transmission

As previously discussed, the cornea is the most sensitive tissue of the entire body. It is rich in low-threshold mechanoreceptors and high-threshold nociceptors. These allow signal transduction for pain, itch, thermal changes, and chemical irritants through multiple rapid ion channels. The signal passes through the trigeminal nucleus and higher pain centers. Adaptation and sensitization processes have been described and characterized in different chronic conditions. Namely, hyperalgesia and allodynia may occur after injury or surgery, because of pathological lowering of sensitivity threshold [10,35].

#### 2.3.4. Immune Regulation

The interplay between the neural and immune systems is an area of intensive research. Nerve fibers can directly interact with resident neutrophils, dendritic, and mast cells, by modulating inflammation, cell migration, and healing responses [36]. Neuromediators can suppress excessive immune activity and limit fibrosis, while pathological denervation can promote inflammatory cytokines [36,37]. The neuro-immune crosstalk on the ocular surface is crucial in chronic inflammatory disease, opening potential therapeutic targets [38].

### 2.4. Pathological Consequences of Nerve Dysfunction

Ocular nerve dysfunction characterizes a broad spectrum of vision-threatening clinical diseases. The pathological loss or reduction in sensory, motor, or autonomic (altogether or heterogeneously combined) nerve fibers adversely affects homeostasis and mechanisms of repair and recovery on the ocular surface [8,10,15].

#### 2.4.1. Neurotrophic Keratopathy

**Figure 1:**
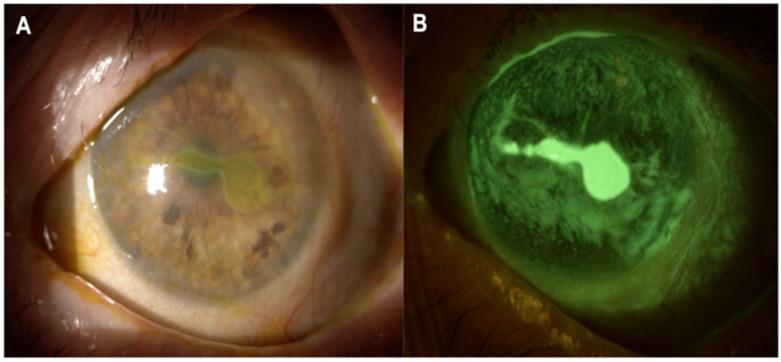
Slit lamp photography (**A**) and fluorescein staining (**B**) of a neurotrophic keratopathy. The margins are well defined, neovascularization is present in the superior quadrant, and there are no signs of infiltrates.

The loss (or significant damage) of corneal sensory innervation culminates in neurotrophic keratopathy (NK). The serious damage to corneal innervation frequently follows herpetic infection, surgical trauma, or nerve compression disorders [39]. The clinical classification of NK has three stages: in stage I, there are superficial punctate epithelial erosions, associated with reduced sensitivity; in stage II, there are persistent and non-healing epithelial defects; stage III culminates with corneal ulcers and stromal melting, characterized by poor prognosis and high risk of perforation and permanent vision loss [40] (Figure 1).

NK arises from a severe deficiency of trophic neuromodulators, such as NGF and substance P, leading to diminished epithelial cell migration, alteration in tight junction integrity, and, consequently, reduced ability to restore corneal epithelium integrity [41]. Along with epithelial defects and reduced healing potential, there is an increased risk for secondary infections.

Recent imaging studies using IVCM revealed dramatic reductions both in nerve density and sub-basal plexus morphology [42]. Furthermore, molecular analyses showed downregulated NGF, along with increased markers of apoptosis. These data highlight a poor regenerative potential until nerve supply is restored [35,37].

#### 2.4.2. Infectious Neuropathies

Herpes simplex (HSV) and varicella-zoster virus (VZV) related infections are major causes of corneal nerve damage, inducing acute and chronic neuropathic syndromes. The neurotropic viral replication and the associated immune activation cause axonal damage and loss of physiological innervation, producing symptoms of pain and chronic numbness, paving the way for herpetic NK [43]. Advanced in vivo imaging shows widespread disruption of nerve architecture [44]. Pain management may require adjunctive therapies such as oral neuropathic pain modulator agents [45].

#### 2.4.3. Surgical Injury

Refractive surgical procedures (PRK, SMILE, and, particularly, LASIK) intrinsically damage corneal nerves, and ultimately decrease sub-basal nerve density and affect corneal sensitivity for months to years, sometimes with severe dry eye symptoms. Namely, up to 80% of LASIK patients experience transient dry eye symptoms, while a remarkable subset has chronic symptoms lasting up to 24 months after surgery. Nerve regeneration rates vary by technique, with SMILE being generally considered more nerve-sparing compared to LASIK [46,47].

#### 2.4.4. Systemic Neuropathies

Systemic disease can affect corneal nerves. Diabetes mellitus is a frequent cause of ocular nerve dysfunction. Diabetic neuropathy arises from chronic hyperglycemia, with accumulation of advanced glycation end-product (AGE), related microvascular ischemia, and chronic low-grade inflammation. Diabetic patients progressively develop corneal hypoesthesia, even before neuropathy elsewhere. The progressive loss of nerve density is strongly correlated with diabetes duration and poor glycemic control [48]. Furthermore, neurological disorders (multiple sclerosis, Parkinson’s, and Alzheimer’s disease) have been recently described in relation to ocular surface nerve alterations. IVCM has been shown to serve as a potential tool for early detection of neurodegenerative effects on peripheral nerves [13,49].

#### 2.4.5. Neuropathic and Nociplastic Pain Syndromes

Some patients present with profound and persistent ocular pain/discomfort, with a remarkable mismatch when symptoms are compared to objective findings (i.e., pain with no stain). It is generally considered that with ocular neuropathic pain, it is the corneal nerve, rather than the surrounding tissue, which is affected. Over time, chronic neuropathic pain can induce central sensitization and maladaptive nociceptive processing, which further aggravates ocular symptoms. Ocular neuropathic pain represents a clinical challenge, since standard topical therapies do not change ocular symptoms. Patients’ management requires a multidisciplinary approach and includes the use of systemic neuromodulators. Moreover, proper quantification of ocular symptoms requires the use of ad hoc questionnaires [50,51].

### 2.5. Diagnostic Techniques

A comprehensive assessment of ocular innervation guarantees a timely and accurate diagnosis of ocular nerve dysfunction. Modern ophthalmic diagnostics combine traditional clinical evaluation with novel in vivo imaging and quantitative testing, combined with patient-reported outcome measures.

#### 2.5.1. Clinical Evaluation

Detailed medical and ophthalmic history remains of paramount importance. It should be focused on risk factors (if any), including prior herpetic infections, history of refractive surgery, diabetes, trauma, or other systemic neuropathies or CNS diseases. Clinical examination includes slit-lamp bio-microscopy, aiming for any sign of epithelial suffering, tear film instability, or reduced blink rate. Testing corneal sensitivity may provide important data for a comprehensive corneal assessment [52].

Pain evaluation relies on validated questionnaires (Ocular Surface Disease Index (OSDI), Neuropathic Pain Symptom Inventory (NPSI), Ocular Pain Assessment Survey (OPAS), or Visual Analogue Scale (VAS)) in order to distinguish neuropathic pain from other etiologies. Dryness, burning, and/or foreign body sensation may have neurogenic causes, rather than inflammatory or tear film etiologies [53,54].

#### 2.5.2. In Vivo Confocal Microscopy (IVCM)

IVCM allows in vivo representation of corneal nerves, as it provides high-resolution of epithelial, subbasal, and stromal nerve fibers with micron-level precision. Morphometric analyses may include nerve fiber length, density, tortuosity, branching, and bead formation. IVCM allows detection of early signs of preclinical nerve loss in diabetes and after refractive surgery, as well as the assessment of inflammation-related changes following herpetic or autoimmune disease [12,13]. Quantitative metrics have been proposed, such as the Corneal Nerve Fiber Density (CNFD) and Branch Density (CNBD) [55,56]. These metrics may help with a large-scale standardization of research findings.

#### 2.5.3. Esthesiometry

The Cochet–Bonnet esthesiometer represents the traditional test for corneal sensitivity assessment. The Cochet–Bonnet esthesiometer consists of a variable-length nylon filament, which is progressively shortened according to the sensitivity. It gently touches the central cornea until the patient perceives sensation. The mechanical threshold value, which varies according to the length of the filament itself, is then recorded and compared with the patient’s age, surgery history, and disease [57]. Novel non-contact and digital esthesiometers now enable measurement of responses to thermal and chemical stimuli, allowing more precise corneal mapping [58,59]. Recent insights have provided correlations between reductions in esthesiometry scores and in vivo loss of nerve fibers [60].

#### 2.5.4. Advanced Ocular Imaging and Testing

Anterior segment optical coherence tomography (AS-OCT) has progressively evolved, implementing corneal nerve mapping and characterization. High definition OCT algorithms allow non-invasive monitoring of nerve proximity, thickness, and surrounding vasculature, with usefulness in assessing recovery after either trauma or surgery [61]. Electrophysiological techniques have offered insights into the functional status of the nerves and their related conduction, from the ocular surface to the central nervous system, although their use remains confined to research settings [62].

Furthermore, cutting-edge research has offered molecular markers of corneal nerve health. The protein or gene expression levels of NGF and BDNF in the tear fluid, as well as the evaluation of inflammatory cytokines and serum autoantibodies, are associated with the severity and regenerative capacity of affected nerves [63,64].

#### 2.5.5. Patient-Reported Outcome Measures and Related Questionnaires

Questionnaires aiming to quantify the severity of dry eye symptoms, pain, discomfort, photophobia, and vision quality are fundamental to correlate objective findings with subjective symptoms. Statistically robust tools (OSDI, OPAS, DEQ-5, and SF-36) facilitate research studies and support direct therapeutic decisions [53].

### 2.6. Therapeutic Strategies and Future Directions

Therapeutic approaches for ocular nerve dysfunction try to restore the original bio-architecture of corneal nerves by restoring physiological molecular signaling. Modern strategies aim to fill the gap between molecular biology, immunology, and bioengineering.

#### 2.6.1. Neurotrophic Factors and Pharmacological Interventions

The use of neurotrophic agents (e.g., the use of recombinant human nerve growth factor) has changed the paradigm in the therapy of neurotrophic keratopathy. Phase III studies have shown the potential of topical cenegermin by promoting corneal nerve regrowth, as well as epithelial integrity and healing of corneal epithelial defects [65]. Studies are also focusing on similar neurotrophic factors (such as BDNF, citicoline, and peptide mimetics), targeting complementary mechanisms of nerve growth and ocular surface repair. Specifically, these agents activate downstream signaling cascades, promoting cellular migration, proliferation, and the synthesis of proteins essential for gap junctions’ integrity [41,66]. Pharmacologic treatment of neuropathic pain (gabapentinoids and tricyclic antidepressants) alleviates ocular symptoms [67].

#### 2.6.2. Regenerative Medicine and Tissue Engineering

Limbal epithelial stem cells, bone marrow-derived stem cells, and induced pluripotent stem cells (iPSCs) have shown efficacy in preclinical studies in restoring nerve architecture and corneal surface regularity [68,69,70]. Bioengineered matrices, like hydrogel scaffolds, collagen-based polymers, and bioprinted nerve guides, can support and drive axonal growth with the intrinsic three-dimensional structure [71,72,73].

#### 2.6.3. Surgical Innovations

Laser refractive techniques are known to damage ocular surface nerves. Refractive techniques have been progressively refined to minimize nerve damage. Of note, SMILE represents the most nerve-sparing alternative nowadays [46]. In addition, corneal neurotization-a surgical technique where healthy peripheral nerves are transferred to the denervated cornea has been gaining momentum, and preliminary results are promising [74].

#### 2.6.4. Neuro-Immune Modulation

Therapies are now focusing on targeting and modulating neuro-immune interactions, especially through the use of peptide antagonists, cytokine inhibitors, and ad hoc delivery of anti-inflammatory molecules. This approach can potentially revolutionize the management of chronic inflammatory and allergic ocular surface conditions, where nerve-immune dysregulation exacerbates disease severity and its related perception [75,76,77,78,79,80].

## 3. Tissue Remodeling Following Nerve Injury

### 3.1. Background

#### 3.1.1. Tissue Homeostasis, Para-Inflammation, and Inflammageing

The ocular surface exists in a dynamic balance between homeostasis and para-inflammation, and when this balance is disrupted, tissue remodeling processes occur as a protective and regenerative response that might evolve toward a correct physiological reconstruction (physiological remodeling) or toward fibrosis and loss of stromal architecture (dysfunctional remodeling) [81,82,83]. At a fundamental level, the term “tissue homeostasis” defines an old concept regarding the maintenance of the morphological, structural, and functional integrity of a tissue/organ; at the cellular level, this aspect implies a cellular homeostasis assuring the intracellular functional balance and a tissue homeostasis guaranteeing the satisfactory balance between proliferation, differentiation, and apoptosis of cellular components. In recent years, the scientific interest has moved toward a better characterization of para-inflammation, inflammation, and the more recent defined inflammageing. While inflammation is an acute innate and immune-mediated inflammatory response to physical, chemical, and biological stressors, para-inflammation is a long-lasting and low-grade inflammation keeping the tissue reactive to eventual stressors (subclinical level) that might evolve in inflammageing under the effect of aging and/or age-related diseases [84,85]. The mechanism appears more complex following nerve damage at the ocular surface, as repair requires proper corneal innervation under a tight interplay between neurogenic and inflammatory soluble mediators attempting to restore homeostasis through a balanced para-inflammation, thus avoiding dysfunctional fibrosis [83,84]. The transition from para-inflammation to chronic inflammation leading to fibrosis, together with local “inflammageing”, may intensify progressive structural and functional impairment of the ocular surface [84,85].

#### 3.1.2. Neurogenic Inflammation and Tissue Remodeling

The corneal region of the ocular surface unit is one of the most densely innervated tissues, and corneal nerve fibers play a pivotal role in maintaining homeostasis through sensory perception, induction of the lacrimal reflex, blinking activation, and release of trophic factors essential for epithelial integrity [1,86]. Any insult or damage to corneal nerves (disease, trauma, or surgical procedures) can lead to reduced corneal sensitivity, epithelial defects, and, in severe cases, irreversible visual impairment [83,86]. These events may evolve differently depending on genetic and epigenetic background, as described in two disabling ocular surface diseases, keratoconus (KC) and ocular cicatricial pemphigoid (OCP), both deeply affecting patients’ quality of life [87,88,89]. Although similar in the mechanism occurring at the corneal epithelial–stromal and basement levels, both chronic inflammatory disorders display different spatial–temporal events leading to physiological interplay between epithelial, stromal layers, and basement layers. In KC, the chronic inflammatory events lead to the long-lasting disruption of the nerve network, including at the sub-basal nerve plexus, the decreased nerve density with concomitant changes in the nerve morphology (increased nerve branching or thickening), and the final reduction in the neurotrophic support (NGF) [87,88]. In the autoimmune-based OCP, chronic and neuroinflammatory events occur starting at the level of the basement membrane (polyvalent Ig-GAM attaches), followed by complement activation and cytotoxic mediator release, leading to the chronic inflammatory events localized at the stromal level (corneal fibroblast activation) [88,89]. Once the disease reaches the level of well-established fibrosis, a reduced density of nerve networking with associated severe reduction in neurotrophic factors can be observed. Although different, these spatial–temporal sequela of events display some interesting remodeling aspects (Table 1) as described and discussed below.

### 3.2. Mechanisms of Action

#### 3.2.1. Keratoconus Remodeling

Remodeling in KC is dominated by structural loss and biomechanical weakening rather than organized healing. This mechanism is characteristic of KC, where nerve injury induces complex cellular changes with keratocyte activation and differentiation into fibroblasts and myofibroblasts producing matrix metalloproteinases (MMPs) and extracellular matrix (ECM) components in an attempt to repair damaged tissue, often resulting in tissue degradation rather than functional scarring [87,90]. While a single mechanism has not been identified so far regulating corneal nerve injury in keratoconus, this is probably the consequence of a complex interplay of multiple causes. Among these, chronic use of contact lenses, intense rubbing, and chronic ocular surface inflammation can all play a role. Nerve regeneration in KC is injury-specific and associated with inflammation, altered expression of neurotrophic factors (including NGF and substance P), and disrupted epithelial–stromal communication, all of which contribute to impaired healing and disease progression [91,92,93]. In this context, the interplay between neurogenic and inflammatory mediators drives changes in corneal structure and sensitivity. Nerve damage triggers neurogenic inflammation mediated by neurotransmitters and neuropeptides, characterized by edema, pain, leukocyte recruitment, and release of inflammatory mediators that further damage nerve fibers [84,94]. Neurotrophic factors such as NGF, TGF-β1, and VEGF act as key regulators of this process, functioning as major profibrogenic and angiogenic mediators interacting with immune and stromal cells and promoting keratocyte activation and myofibroblast differentiation [91,95]. Stromal remodeling is particularly evident in KC, which was historically considered a non-inflammatory disorder but is now recognized as a condition associated with chronic low-grade inflammation in genetically predisposed individuals exposed to repeated microtrauma [90,96]. Transcriptomic analyses of KC corneal specimens have demonstrated overexpression and positive correlation between TGF-β1 and α-SMA, indicative of a contractile/fibrotic phenotype, together with a shift from keratocan to procollagen production, pathological ECM accumulation, and impaired matrix turnover [87,97]. In parallel, inflammatory markers such as ICAM-1, IL-10, and HLA-DR are upregulated, supporting the presence of chronic inflammation [96,98]. The resulting remodeling outcome in KC is progressive stromal thinning, loss of biomechanical rigidity, and ectatic protrusion, representing a non-reparative process not primarily characterized by fibrosis. KC tissue remains continuously weakened and is not replaced by functional scar tissue, with cicatrization occurring only focally and late in the disease course, mainly at the level of Bowman’s membrane [87,98,99].

#### 3.2.2. Ocular Cicatricial Pemphigoid Remodeling

Tissue remodeling in OCP develops within a chronic autoimmune inflammatory background characterized by overt fibrosis. Studies of the OCP microenvironment show that neurogenic inflammation plays a central role in tissue damage and repair [88,100]. Increased α-SMA expression in conjunctival biopsies, particularly in advanced disease stages, reflects myofibroblast activation, fibrosis progression, and loss of ocular surface functionality [100,101]. Transcriptomic analyses reveal upregulation of fibrosis-related genes and ECM components such as COL1A1, COL3A1, and fibronectin, alongside downregulation of genes related to ocular surface function, including goblet cell and epithelial differentiation markers [101,102]. ECM reorganization in OCP is mediated by MMP-1 and MMP-9, with dysregulation of their tissue inhibitors (TIMPs), resulting in proteolytic imbalance, keratocyte apoptosis, stromal disorganization, and Bowman’s membrane alterations [100,103]. These processes are driven by both innate immune cells (mast cells, eosinophils, macrophages) and adaptive immune cells (T and B lymphocytes) infiltrating the conjunctiva [88,104]. OCP is characterized by autoantibodies against basement membrane proteins (BP180, BP230, laminin-332), chronic subepithelial inflammation, fibroblast activation, excessive collagen deposition, and cicatricial fibrosis leading to fornix shortening, symblepharon, severe dry eye, and secondary corneal opacification [88,89,105]. Fibrosis represents the key driver of disease progression and visual loss.

### 3.3. Future Perspectives

#### 3.3.1. The Dual-Aspect of Remodeling and Informations

Neuronal involvement in tissue remodeling is fundamental, encompassing developmental pruning, adult plasticity, and injury repair, with neurons and glial cells interacting dynamically with the ECM to guide regeneration and modulate inflammation [106]. In KC, corneal nerves become thickened and displaced anteriorly, interacting with stromal keratocytes and influencing their differentiation into myofibroblasts through growth factors such as NGF, thereby contributing to ECM remodeling, stromal thinning, and opacity [91,93,107]. In OCP, NGF plays a central modulatory role in inflammation and fibrosis through its receptors TrkA and p75NTR, influencing fibroblast activation, cytokine release (including TGF-β), and potentially myofibroblast apoptosis in a context-dependent manner [95,100,108]. Neuronal–immune–fibroblast crosstalk sustains chronic inflammation and aberrant ECM deposition, ultimately driving scarring and visual impairment [100,108].

#### 3.3.2. Fibrogenic Profiles and Novel Approaches

Taken together, KC and OCP represent paradigmatic but opposite models of maladaptive tissue remodeling. Understanding the molecular mechanisms underlying these processes is crucial for the development of targeted therapies aimed at controlling inflammation, preventing fibrosis, and promoting neuroprotection.

## 4. Therapeutic Potential of Hemocomponents in Neuronal and Cicatricial Pathologies

### 4.1. Background

Blood-derived eye drops have been increasingly adopted as biological therapies for severe ocular surface disease (OSD), particularly when epithelial instability, neurotrophic impairment, and chronic inflammation coexist, and conventional treatments prove insufficient. Accordingly, these preparations are best interpreted as “biological formulations” that provide a tear-mimetic and trophic microenvironment supporting epithelial repair, immune balance, and neural recovery, rather than as single-factor drugs. Their activity arises from the coordinated action of multiple components naturally pooled by human physiology [7,109,110].

#### 4.1.1. What Are EDHO (Eye Drops of Human Origin) and Why Do They Matter?

An important conceptual and regulatory evolution in this field is the recognition that serum eye drops represent only one member of a broader family of Eye Drops of Human Origin (EDHO) to encompass diverse human-derived ocular formulations beyond classical serum, including platelet-derived products and cord blood-derived preparations, while explicitly acknowledging persistent heterogeneity in production standards and clinical indications [111]. This broader definition is clinically relevant because different preparations deliver distinct biologically active profiles, and may therefore be better suited to different phenotypes of disease (e.g., predominantly neurotrophic vs. inflammatory vs. cicatricial).

#### 4.1.2. Composition of Blood-Derived Products

From a biological standpoint, blood-derived eye drops act as multicomponent reservoirs. Their potential relevance for ocular surface repair is linked to the presence of the following:

- *Growth factors and neurotrophins.* This includes EGF, IGF-1/2, FGF, PDGF, VEGF, and neurotrophic mediators such as NGF, supporting epithelial migration/proliferation and neural maintenance.
- *Cytokines and immunomodulatory mediators*. This balance of pro- and anti-inflammatory signals (e.g., IL-1 family and IL-6, IL-10) may contribute to restoring immune homeostasis, with effects influenced by concentration and baseline inflammatory milieu [7,109,110].
- *Antimicrobials, antioxidants, lipids, and small molecules.* Additional tear-relevant components: Antimicrobial and antioxidant factors and lipid/small-molecule constituents that may support barrier function and tear-film stability in complex OSD phenotypes [7,109].
- *Extracellular vesicles and regulatory RNAs.* Emerging mediators (EVs/exosomes and miRNAs) are potentially involved in intercellular signaling relevant to wound healing and fibrosis [112].

#### 4.1.3. Why “Physiological Cocktails” May Outperform Single Molecules

A recurring challenge in ocular surface therapeutics is that single-molecule approaches rarely address the full complexity of epithelial repair in inflamed or denervated tissue. Corneal homeostasis and regeneration require synchronized signals controlling proliferation, migration, differentiation, immune tolerance, and matrix remodeling. Blood-derived products provide parallel signaling inputs that may better reflect this biology: rather than attempting to replace one missing mediator, they offer a coordinated mixture that can engage multiple pathways simultaneously [7,109,110]. This is particularly relevant in neurotrophic and cicatricial disease, where epithelial failure is typically not monofactorial.

At the same time, the “cocktail advantage” has a practical corollary: composition matters. Dose–response relationships may not be linear, and certain factors can become inhibitory at higher concentrations in specific cellular contexts, showing that increasing EGF concentrations can downregulate clonal growth in vitro, supporting the principle that “more is not always better,” and that formulation and dilution should be rationalized [110].

#### 4.1.4. Comparison of Key Preparations

- *Autologous serum eye drops (ASED).* ASED are typically prepared from the patient’s peripheral blood and are widely used across severe dry eye, persistent epithelial defect, corneal ulceration, neurotrophic keratopathy, and other complex OSDs [7,109,110,113]. Strengths include immunologic compatibility and a tear-mimetic composition; limitations include inter-individual variability, logistical burdens, and limited harmonization across centers [111].
- *Platelet-rich plasma (PRP) and platelet lysate (PL).* Platelet-derived formulations provide a higher reservoir of platelet-released mediators (e.g., PDGF and TGF-β family) and may be advantageous in refractory epithelial defects and postoperative surface instability [114,115]. PL, in particular, has been studied as a standardized, efficient source of growth factors for ocular surface applications, with translational interest in quality consistency and potency [115]. However, protocol heterogeneity (activation methods, leukocyte content, and storage) complicates cross-study comparisons and supports the need for shared minimal quality parameters [111].
- *Cord blood–derived products.* Allogeneic preparations are proposed when autologous approaches are not feasible or when a distinct trophic/immunomodulatory profile is desired [110,116]. These products require robust donor screening, traceability, and governance aligned with SoHO principles [111].

#### 4.1.5. EDHO Within the New EU SoHO Regulatory Framework

The regulatory landscape in Europe is evolving with direct implications for EDHO. In 2024, the EU adopted a new Regulation on standards of quality and safety for substances of human origin (SoHO) intended for human application [117].

This framework aims to strengthen safety, traceability, and governance for both autologous and allogeneic pathways, while accommodating emerging applications such as human-derived eye drops [118]. For clinical translation, this reinforces the need for clearer product definitions, shared minimal quality parameters (including microbiological safety), and harmonized vigilance systems [111].

### 4.2. Mechanisms of Action in Neurotrophic and Cicatricial Disease

The therapeutic effects of blood-derived eye drops in ocular surface disease derive from their ability to simultaneously modulate epithelial repair, inflammatory balance, and neural regeneration. Unlike conventional topical therapies targeting isolated pathways, these biological formulations act on multiple levels of tissue homeostasis, reflecting the multifactorial nature of neurotrophic and cicatricial disorders.

#### 4.2.1. Promotion of Epithelial Migration and Proliferation

One of the most consistent biological effects of blood-derived products is the stimulation of corneal epithelial migration and proliferation. Growth factors such as epidermal growth factor (EGF), insulin-like growth factors (IGF-1 and IGF-2), fibroblast growth factors (FGFs), and platelet-derived growth factor (PDGF) are central mediators of epithelial wound closure and stratification. These molecules regulate cytoskeletal reorganization, cell–cell adhesion, and integrin-mediated attachment to the extracellular matrix, processes that are critically impaired in neurotrophic and post-inflammatory conditions [109,119,120].

Experimental data derived from in vitro and ex vivo corneal models have shown that epithelial cell responses to growth factors are highly dose dependent. Notably, increasing concentrations of EGF beyond physiological levels may paradoxically reduce epithelial clonogenic capacity and impair organized cell migration, highlighting the importance of balanced trophic signaling rather than supraphysiological stimulation. This concept supports the biological rationale for using blood-derived products, in which growth factors are delivered within a physiologic range and in combination with complementary mediators rather than as isolated recombinant molecules.

#### 4.2.2. Modulation of Inflammation and Immune Responses

Beyond epithelial regeneration, blood-derived eye drops exert significant immunomodulatory effects. Inflammatory cytokines and chemokines present in controlled concentrations—together with anti-inflammatory mediators such as IL-10, transforming growth factor-β (TGF-β), and soluble growth factor receptors—contribute to restoring immune homeostasis at the ocular surface. This balanced inflammatory milieu is particularly relevant in chronic ocular surface diseases, where persistent low-grade inflammation perpetuates epithelial damage and nerve dysfunction.

Furthermore, antimicrobial peptides (e.g., defensins and cathelicidins) and innate immune components contribute to microbial defense without inducing epithelial toxicity, distinguishing blood-derived formulations from conventional antiseptic or antibiotic approaches. The combined presence of these mediators supports the concept of a “physiological immunomodulation” rather than pharmacological immunosuppression, aligning with the goal of restoring tissue equilibrium rather than merely suppressing inflammation [109,121,122].

#### 4.2.3. Support of Nerve Regeneration and Neurotrophic Function

Neurotrophic support represents a key mechanism underlying the clinical efficacy of blood-derived products in neurotrophic keratopathy and other nerve-related ocular surface disorders. Neurotrophins such as nerve growth factor (NGF), along with IGFs and other signaling molecules, promote axonal survival, sprouting, and functional recovery of corneal sensory nerves. Experimental and clinical studies have demonstrated improvements in corneal sensitivity and subbasal nerve morphology following treatment with serum- or platelet-derived formulations, supporting their role in neural repair [80,123,124].

Importantly, neural recovery is tightly coupled to epithelial health: reinnervation enhances epithelial metabolism and wound healing, while restored epithelial integrity provides trophic support to regenerating nerves. This bidirectional interaction reinforces the concept that effective therapy must address both epithelial and neural compartments simultaneously.

#### 4.2.4. Dose- and Formulation-Dependent Biological Effects

Accumulating evidence indicates that the biological activity of blood-derived products is influenced not only by qualitative composition but also by concentration, preparation method, and dosing regimen. Growth factor effects are dose dependent, supporting the need for rational formulation and dilution strategies rather than overphysiological stimulation [125].

Differences in preparation methods (serum vs. platelet-rich plasma vs. platelet lysate; fresh vs. frozen formulations) influence the availability of bioactive mediators and may contribute to variable clinical responses. In practice, formulation selection is phenotype-driven rather than interchangeable. For instance, diluted autologous serum (commonly 20–40%) is frequently employed in inflammatory-dominant severe dry eye disease due to its tear-mimetic composition and relatively balanced cytokine profile [7,126]. In contrast, platelet-rich preparations, characterized by higher concentrations of platelet-derived growth factors such as PDGF and TGF-β family members, have been investigated in persistent epithelial defects and moderate-to-severe neurotrophic keratopathy, where enhanced epithelial proliferation and trophic support are required [6,110]. In cicatricial disease, these formulations are generally used as adjuncts to systemic immunomodulation to support epithelial integrity while the underlying autoimmune process is controlled [126]. This approach reflects structured, mechanism-based clinical decision-making rather than uniform application across conditions. A practical comparison between serum-based and platelet-based EDHO, including preparation principles, biological profiles, and phenotype-oriented clinical selection, is summarized in Table 2.

### 4.3. Clinical Applications and Efficacy

#### 4.3.1. Indications

Blood-derived eye drops have found their principal clinical application in conditions characterized by impaired epithelial healing, altered corneal innervation, and chronic inflammation.

Among these, neurotrophic keratopathy (NK) represents the most paradigmatic indication, where impairment of trigeminal innervation leads to corneal epithelial breakdown, impairment of healing, and development of corneal ulceration, melting, and perforation. Several clinical studies have demonstrated that autologous serum and platelet-derived formulations can promote epithelial closure, improve corneal sensitivity, and reduce the risk of stromal involvement in patients with moderate to severe NK [6,83,127].

Beyond classical neurotrophic disease, severe dry eye disease (DED) has emerged as a major indication for blood-derived eye drops. In particular, DED caused by certain etiological drivers, particularly in its inflammatory and mixed neurotrophic phenotypes, can benefit from anti-inflammatory therapies, T-cell immunomodulatory drugs, in addition to biologic tear substitutes such as autologous serum and platelet-rich plasma. In these patients, conventional tear substitutes often fail to restore ocular surface homeostasis, whereas serum- or platelet-based therapies provide trophic and immunomodulatory support that translates into improved symptoms and dry eye signs, such as Schirmer test results, TBUT, and corneal and conjunctival staining measurements [110,126,128,129].

Blood-derived products have also shown benefit in chemical and thermal ocular injuries, where rapid restoration of the epithelial barrier and suppression of inflammation are critical to preventing cicatricial sequelae. Clinical studies and case series report accelerated wound healing in patients with persistent epithelial defects and improved structural outcomes when these therapies are integrated early into standard medical treatments [130].

Similarly, post-surgical and post-radiation ocular surface damage, including that following keratoplasty, refractive surgery, or orbital radiotherapy, may benefit from blood-derived products through enhanced epithelial recovery and modulation of chronic inflammatory responses [131,132,133]. Some studies reported improvement in DED symptoms, corneal staining measurements, conjunctival hyperemia, and ocular surface inflammation in patients treated with blood-derived products after refractive surgery [131,132]. Moreover, several studies reported a significant reduction in neuropathic corneal pain following treatment with blood-derived eye drops, which was associated with improvements in sub-basal nerve plexus parameters [134].

#### 4.3.2. Evidence of Efficacy

- *Timing, Dosing, and Treatment Duration.* The clinical efficacy of blood-derived eye drops is strongly influenced by the timing of initiation, dosing frequency, and treatment duration. Evidence suggests that early intervention, particularly during the reversible phases of epithelial and neural dysfunction, is associated with better anatomical and functional outcomes. In contrast, delayed treatment in advanced cicatricial disease may limit the regenerative potential of these therapies.

Most published protocols report dosing regimens ranging from four to eight instillations per day, tailored according to clinical response to treatment; treatment durations vary from several weeks to several months, depending on disease severity and response [134]. Importantly, both experimental and clinical data indicate that the biological effects of blood-derived products are dose-dependent but not linearly proportional. Excessive concentrations of certain growth factors, notably EGF, may paradoxically impair epithelial proliferation or alter differentiation patterns, underscoring the importance of balanced formulations and personalized dosing strategies [125].

- *Clinical Evidence and Real-World Experience.* The clinical efficacy of blood-derived eye drops is supported by a growing body of prospective and retrospective studies, as well as real-world observational data. Autologous serum eye drops have consistently demonstrated benefits in improving corneal epithelial integrity, reducing ocular discomfort, and enhancing visual function in patients with severe ocular surface disease [6,127,128]. An American Academy of Ophthalmology Preferred Practice assessment report reviewed 10 studies and found 8 to be high quality, demonstrating improved symptoms and at least one objective clinical sign of dry eye (i.e., ocular surface staining, Schirmer testing, tear film breakup time, or cytologic analysis) with the use of serum tears. Moreover, a task force report from the European League Against Rheumatism (EULAR) provided an algorithm for treating ocular dryness and recommends the use of autologous serum tears in patients whose symptoms were uncontrolled after ocular lubricants and CsA [126].

Plated derived preparation, such as platelet-rich plasma and platelet lysate preparations, have shown comparable or superior outcomes in selected patient populations, particularly those with refractory epithelial defects or pronounced neurotrophic impairment [110,125,126,129]. Indeed, these preparations differ from autologous serum tears in that they contain clotting proteins and biologically active agents derived from platelets, such as epidermal growth factor, transforming growth factor–β, platelet-derived growth factor, NGF, and insulin-like growth factor [126]. Several studies have shown that platelet-derived preparations used in patients with persistent epithelial defects (PED) or severe dry eye disease (DED) secondary to Sjögren’s syndrome or graft-versus-host disease, who were unresponsive to conventional therapies, resulted in significant improvement in dry eye symptoms, particularly photophobia, as well as in objective clinical parameters, including tear break-up time and fluorescein corneal staining. These benefits were accompanied by faster corneal and conjunctival epithelial healing, improved corneal clarity, and better best-corrected visual acuity [110].

Importantly, accumulated clinical experience indicates that therapeutic response is influenced by both disease phenotype and product characteristics. Patients with predominantly neurotrophic or trophic deficiency appear to derive the greatest benefit, whereas inflammatory-dominant phenotypes may require combination approaches. These observations reinforce the concept that blood-derived products should be integrated into a personalized, mechanism-based treatment strategy.

### 4.4. Practical Considerations and Unmet Needs

Despite the growing body of evidence supporting the clinical efficacy of blood-derived eye drops, their broader implementation in routine ophthalmic practice remains challenged by several practical and regulatory issues. These limitations are largely related to heterogeneity in product preparation, lack of standardized quality parameters, logistical constraints, and disparities in regulatory frameworks across countries [7].

#### 4.4.1. Variability in Preparation and Regulatory Heterogeneity

One of the major limitations in the clinical translation of blood-derived eye drops is the marked variability in preparation protocols. Differences in blood collection methods, centrifugation protocols, use of anticoagulants, activation procedures, dilution ratios, and storage conditions result in products with substantially different biological compositions. This variability affects not only growth factor and cytokine concentrations but also the presence of extracellular vesicles, residual cellular components, and antimicrobial peptides, ultimately influencing clinical efficacy and reproducibility.

Regulatory approaches to blood-derived eye drops also differ substantially among countries. While some national frameworks classify these products as medicinal products, others regulate them under transfusion or tissue-and-cell legislation [117,135]. The recent European Union Regulation on Substances of Human Origin (SoHO) aims to harmonize standards related to safety, traceability, and donor protection, while explicitly acknowledging emerging products such as Eye Drops of Human Origin (EDHO) [118]. This framework represents an important step toward a risk-based, proportionate regulatory model that balances innovation with patient safety.

#### 4.4.2. Storage, Stability, and Delivery Challenges

Another critical aspect concerns the stability and handling of blood-derived eye drops. Most formulations require cold storage or freezing to preserve biological activity, which imposes logistical constraints for both healthcare facilities and patients. Repeated freeze–thaw cycles may alter protein conformation and reduce bioactivity, while inadequate storage conditions increase the risk of microbial contamination.

Recent efforts to improve stability—such as lyophilization or optimized freezing protocols—have shown promise in extending shelf life and simplifying transport and storage. However, these approaches introduce additional variables that may influence biological activity and therefore require careful validation. Optimizing delivery systems, including single-dose packaging and standardized reconstitution protocols, remains a key challenge for ensuring safety, reproducibility, and patient adherence [136].

#### 4.4.3. Need for Standardization and Biomarkers of Response

A major unmet need in the field is the lack of standardized criteria to guide product selection, dosing regimens, and treatment duration. Currently, therapeutic decisions rely largely on empirical experience rather than evidence-based algorithms [7].

Equally important is the absence of validated biomarkers capable of predicting or monitoring therapeutic response. Potential candidates include tear film inflammatory mediators, growth factor profiles, corneal nerve parameters assessed by in vivo confocal microscopy, and molecular indicators of epithelial regeneration. The identification and validation of such biomarkers would represent a critical step toward personalized use of blood-derived therapies.

#### 4.4.4. Cost, Accessibility, and Health System Integration

Cost and accessibility remain significant barriers to the widespread adoption of blood-derived eye drops. The need for specialized facilities, trained personnel, and regulated production environments contributes to variability in availability and reimbursement across healthcare systems. From a public health perspective, harmonization of regulatory frameworks, combined with robust clinical and economic evidence, will be essential to support broader integration of EDHO into standard ophthalmic care pathways [126].

### 4.5. Future Perspectives

#### 4.5.1. Toward Precision Medicine in Ocular Surface Disease

The increasing complexity of ocular surface disorders, particularly those characterized by neurotrophic and inflammatory components, highlights the need for a shift from empirical treatment strategies toward a precision medicine–based approach. Blood-derived eye drops, by virtue of their biological complexity, represent an ideal platform for such evolution. Rather than being used as uniform therapeutic agents, these products could be tailored according to patient-specific pathological mechanisms, including the relative contribution of neurodegeneration, inflammation, epithelial instability, and immune dysregulation [137].

Advances in phenotyping techniques—such as in vivo confocal microscopy, tear proteomics, and molecular profiling—are progressively enabling the identification of distinct disease endotypes. Stratifying patients based on neuro-inflammatory signatures may allow clinicians to select the most appropriate blood-derived formulation, optimize dosing regimens, and predict therapeutic response [7,109]. In this context, the integration of biomarker-driven approaches represents a key step toward personalized management of complex ocular surface disease.

#### 4.5.2. Combination Therapies and Multimodal Approaches

Given the multifactorial nature of ocular surface disease, blood-derived eye drops are often integrated into multimodal treatment strategies rather than used as isolated interventions. In clinical practice, combination approaches typically involve the association of serum- or platelet-based formulations with targeted anti-inflammatory therapy (e.g., short courses of topical corticosteroids during active inflammation or calcineurin inhibitors in immune-mediated dry eye), particularly in inflammatory-dominant phenotypes. In advanced neurotrophic keratopathy or persistent epithelial defects, blood-derived eye drops may also be combined with regenerative procedures such as amniotic membrane transplantation to enhance epithelial recovery and modulate stromal remodeling [109,126].

In cicatricial disorders, integration with systemic immunomodulatory therapy is often required to control the underlying immune process while supporting surface repair. These examples illustrate that blood-derived products are most effective when incorporated into a structured, mechanism-based treatment plan tailored to disease stage and phenotype [138].

#### 4.5.3. Regulatory Harmonization and Future Directions

The progressive implementation of the European Regulation on Substances of Human Origin (SoHO) marks a pivotal step toward harmonizing regulatory oversight for blood-derived products across Member States [117,118]. By introducing a unified, risk-based framework, the regulation aims to ensure high standards of safety and traceability while fostering innovation and cross-border collaboration.

For EDHO, this regulatory evolution offers an opportunity to move toward standardized production protocols, shared quality benchmarks, and clearer pathways for clinical adoption. At the same time, it underscores the need for continued dialogue between clinicians, transfusion specialists, regulatory authorities, and industry stakeholders to ensure that innovation remains aligned with patient safety and clinical relevance.

Ultimately, the integration of precision medicine principles, robust regulatory frameworks, and multidisciplinary collaboration will be essential to fully realize the therapeutic potential of blood-derived eye drops. As evidence continues to accumulate, these products are likely to assume an increasingly defined role within personalized treatment algorithms for ocular surface disease.

## 5. Conclusions

The ocular surface emerges from this review as a highly integrated neuroepithelial–immune unit, in which corneal innervation plays a central and non-redundant role in maintaining tissue homeostasis, coordinating repair mechanisms, and modulating inflammatory responses. Neural dysfunction—whether related to disease, injury, or surgery—initiates epithelial and immune alterations that contribute to chronic ocular surface damage, impaired regeneration, and progressive tissue remodeling.

Across different disease models, disruption of neurogenic signaling represents a common pathogenic mechanism linking persistent inflammation, defective healing, and fibrotic changes. Within this context, blood-derived eye drops constitute a biologically coherent therapeutic approach, providing a multifactorial and physiologically balanced repertoire of mediators capable of supporting epithelial repair, modulating inflammation, and promoting nerve recovery.

Future advances will rely on integrating neurobiological insights with standardized regenerative therapies and harmonized regulatory frameworks, enabling more targeted and personalized management of complex ocular surface disorders.

## Figures and Tables

**Table 1:** Cell and matrix comparison between keratoconus and ocular cicatricial pemphigoid.

Disease	Early	Intermediate	Late	Final
**KC**	Mechanical Attack	Stromal reduction	corneal thickness	Apoptosis Matrix degradation Nerve reduction ↓ Loss of tissue function ***degenerative***
**Cell effectors**	Innate immune response; epithelial response (TLRs)	Keratocytes’ activation ↓↑ MMPs	↑ Cell apoptosis	
**stroma**	Keratocytes’ activation	↓ matrix (keratocan; collagen)	↑ cell apoptosis ↑ matrix proteins catabolism	
Biomarkers *	↓ TGFβ1; ↑ IL6; ↑TNFα			
**Therapeutic approach**	Cross-linking			
**OCP**	Autoimmune Attack	Stromal fibrosis	Cicatricial forms	Fibroblast activation Matrix hyperdeposition Nerve alteration ↓ Loss of tissue function due to overt fibrosis ***Remodeling differently***
**Cell effectors**	Innate + adaptive (IgGAM) response Macrophages (M1–M2); Mast Cells, Eosinophils, Neutrophils	↑ myoFBs ↓↑ MMPs	↑ myoFBs ↓ MMPs	
**stroma**	fibroblast activation	↑ collagen ↓↑ MMPs	↑ myoFBs ↑ matrix proteins anabolism	
Biomarkers *	↑ TGFβ1; ↑ IL6; ↑ IL4			
**Therapeutic approach**	Immunosuppression			

Legend: KC, keratoconus; OCP, ocular cicatricial pemphigoid; * representative biomarkers; MMPs, metalloproteinases; myoFBs, myofibroblasts; and IG-GAM, polyvalent immunoglobulins.; ↑/↓, up or down regulation or expression.

**Table 2:** Practical comparison of serum-based and platelet-based Eye Drops of Human Origin (EDHO) in corneal disease. The table summarizes practical differences between serum-based and platelet-based EDHO, highlighting preparation principles, biological profiles, and phenotype-oriented clinical selection.

Feature	Serum-Based EDHO (Autologous Serum Eye Drops, ASED)	Platelet-Based EDHO (PRP/Platelet Lysate)
Source material	Peripheral blood (no anticoagulant; clot formation)	Peripheral blood with anticoagulant → platelet concentration ± activation or lysis
Preparation principle	Clot formation → centrifugation → serum separation → dilution (commonly 20–50%) → sterile aliquoting	Platelet concentration (single/double spin) → activation or freeze–thaw lysis → debris removal/filtration → aliquoting
Biological profile	Tear-mimetic composition; balanced levels of EGF, TGF-β, IGFs, NGF, cytokines	Higher concentration of platelet-derived growth factors (e.g., PDGF, TGF-β family, EGF-related pathways); greater trophic load
Primary biological effect	Restoration of epithelial homeostasis and immune balance	Enhanced epithelial proliferation, stromal support, neurotrophic stimulation
Typical clinical indications	Severe dry eye disease (especially inflammatory-dominant phenotypes), Sjögren’s syndrome, early–moderate neurotrophic keratopathy (NK), persistent epithelial defects (PEDs)	Refractory PED, moderate–severe NK, post-surgical epithelial instability
Clinical rationale for selection	Preferred when tear-substitute-like, immunologically compatible formulation is needed	Considered when stronger trophic stimulation is desired or when inadequate response to serum-based therapy occurs
Dosing in clinical practice	4–8 instillations/day; duration individualized (weeks–months)	4–8 instillations/day; duration individualized
Advantages	Long clinical experience; autologous compatibility; relatively simple preparation	Higher growth factor availability; potential for faster epithelial response
Limitations	Inter-patient variability; logistical burden; limited inter-center standardization	Greater protocol heterogeneity (platelet concentration, leukocytes, activation method); quality control complexity
Theoretical concerns	Excessive concentration (if undiluted) may alter growth factor balance	High TGF-β exposure (protocol-dependent); variability in platelet content

Legend: EDHO: Eye Drops of Human Origin; ASED: Autologous Serum Eye Drops; PRP: Platelet-Rich Plasma; NK: Neurotrophic Keratopathy; PED: Persistent Epithelial Defect. All listed products fall under the EU regulatory framework for Substances of Human Origin (SoHO) and require traceability, sterility assurance, and defined quality control procedures. Variability in preparation protocols (platelet concentration, leukocyte content, activation method, and storage conditions) may influence biological composition and clinical performance.

## Data Availability

No new data were created or analyzed in this study.
